# The impact of COVID-19 social distancing and isolation recommendations for Muslim communities in North West England

**DOI:** 10.1186/s12889-021-10869-8

**Published:** 2021-04-28

**Authors:** Shaima M. Hassan, Adele Ring, Naheed Tahir, Mark Gabbay

**Affiliations:** 1 Department of Primary Care and Mental Health, Institute of Population Health, University of Liverpool, Liverpool, L69 3GL UK; 2 NIHR Applied Research Collaboration NWC, Liverpool, England

**Keywords:** Muslim community, COVID-19, Health inequalities, Psychological distress, Information provision, Religion

## Abstract

**Introduction:**

People from Minority Ethnic backgrounds living in the UK are at greater risk of not only contracting COVID-19, but also experiencing serious consequences of the virus. These emerging health inequalities mirror those already evident in UK society.

**Aim:**

The aim of this study was to understand how COVID-19 and the associated imposed restrictions affected the lives of people from the Muslim community living in the North West of England.

**Method:**

Twenty-five in-depth qualitative interviews and four focus groups (*n* = 22) explored individual experiences of COVID-19 and imposed restrictions. Data were analysed thematically.

**Findings:**

The virus and associated imposed restrictions had negative impacts on the psychological wellbeing of participants, their families and the wider community. Worry and low mood were particular features of participant’s pandemic stories. Main concerns were those of contracting and transmitting the virus to others and employment-related difficulties. Low mood was particularly linked to the impact of restrictions on fundamental interactions embedded within cultural and religious practices. These practices are central to feelings of belonging and connectedness within the Muslim community. Religious beliefs were important in helping to mitigate psychological distress for some participants.

**Conclusion:**

Psychological distress was associated with COVID-19 virus and impact of COVID-19 restrictions on livelihoods and fundamental human interactions. Better provision of culturally appropriate information, improving local channels of communication and practical support are important during times of pandemic when usual support systems may be disrupted.

## Introduction

Differences in mortality from COVID-19 highlight familiar health inequalities for disadvantaged populations and there is growing evidence to suggest that COVID-19 may not only be replicating these inequalities but also exacerbating them [1, 2]. When looking at people of working age (20-64 yrs., reflecting the age range of the current study sample), populations that are predominately Muslim such as people of Bangladeshi or Pakistani ethnicity, had a much higher risk (80 and 50% respectively) of COVID-19 death than people of White British ethnicity [1].

Recent evidence also shows that not only the virus but also the measures taken to contain it, are having a disproportionately negative impact on people from Ethnic Minority groups further widening health inequalities for these groups [1, 3]. Evidence shows that people from Ethnic Minority groups are more likely to work in occupations where they will be in frequent contact with people and consequently more exposed to the virus, with 1 in 5 people in these types of occupations being from Ethnic Minority groups [1].

Furthermore, the recent Muslim census showed that the Muslim community have a higher proportion of keyworkers (those in public or private sector employment providing an essential service, such as health and social care, education and childcare, food and other necessary goods, etc.) with up to 33% male and 37% female compared to the national average of 22% male and 26% female [4]. Data also show that 15% of participants had lost their jobs as a result of COVID-19 and health protection measures. The report raises grave concerns about job losses among Muslims that are proportionally six times the national average. Many Muslims are struggling financially during the COVID-19 pandemic, which will contribute to the negative impact of the pandemic on their mental health and wellbeing [4]. Evidence from the UK charity, Muslim Youth Helpline shows higher levels of depression compared to the general population within Muslim communities during 2019 and a surge of general advice and crisis calls during the first national lockdown [5]. Although religion was not included as a variable in the report, around a third of the UK ethnic minority population are Muslim [6].

The UK government’s response to slow the transmission of the virus commenced in March 2020 with a full lockdown across England. This included closure of all non-essential businesses and schools and the requirement for people to stay at home and only leave for essential purposes including for work (if unable to work from home), for 1 h’s exercise per day and for specific caring responsibilities. Citizens were also required to keep a two metre distance from those outside their household wherever possible and to self-isolate away from others for 2 weeks should COVID-19 symptoms be experienced [7, 8].

Some measures were relaxed in May 2020 with the re-introduction of unlimited outdoor activity, the return of people to workplaces (where COVID-secure) and the gradual reopening of social and recreation spaces [9, 10]. More recently, a three-tier system of restrictions was introduced in October 2020 [11] and a national lockdown introduced in November 2020 [12] followed by a stricter national lockdown ‘Stay at Home’ in January 2021 [13] to address a resurgence in cases.

The current study explored the impact of COVID-19 and restrictions, on Muslims living in the North West of England. This study is part of a wider COVID-LIV Area B study (incorporating a number of qualitative social science sub-studies that are part of the larger COVID-LIV household virology research) exploring perceptions of risk and experiences of the COVID-19 pandemic for households, communities and organisations in one region of North West of England. The current paper reports findings about the impact of COVID-19 and restrictions to slow its spread, on the lives of Muslims in North West England.

## Method

### Study design

This qualitative study design combines one-to-one semi-structured interviews (*n* = 25) and four focus groups (*n* = 22), with members of the Muslim community living within the North West of England. This approach is particularly suited for gaining in-depth insight into the lived experience of study participants.

### Participant recruitment and consent

The sample in this study reflects the sample highlighted in the Muslim community members perception of Covid-19 risk paper [14]. Using purposive sampling, people who are aged 18 years and speak English from the Muslim community living within North West England were invited to take part in the study. Potential participants were invited to participate via established Muslim community groups including, Local Muslim mosque groups (using social media platforms including, WhatsApp groups, Facebook and email), and other local Muslim groups/networks identified by a public adviser [NT] working with the lead researcher [SH] [14]. Information about the study including the contact details of researcher, was circulated via these groups and media platforms.

Potential participants who registered their interest in the study were provided with an electronic copy of the study information sheet and consent form. They were followed up with a phone/zoom call the next day or at a time convenient for them, to discuss the study and what would be involved should they agree to participate.

Participants who agreed to take part in an interview were given the option to do so via telephone or online video call (Zoom) as appropriate for the participant. Potential participants that agreed to take part in a focus group were invited to join one of four scheduled online (via Zoom) focus group meetings [14].

Interviews lasted 30 to 45 min and focus groups 60 to 75 min and they were audio recorded with participants’ permission. Prior to interviews and focus groups, participants were asked to read and sign the study consent form (providing an electronic signature or typing their name into the consent form, or signing and scanning the form) and return it to the research team via email. At the start of each interview and focus group, consent was reviewed and verbal consent recorded.

Audio recordings were transcribed verbatim by a professional transcriber and transcripts were reviewed for accuracy and anonymised by members of the research team prior to importing into NVivo software (qualitative analysis software) to support the management and coding of data [14].

### Data collection

Data collection took place during the initial ‘stay at home’ lockdown period commencing on the 23rd of March and the ‘stay alert’ restrictions imposed on the 11th May. Data collection ended the first week of July 2020.

Data were collected through 25 one-to-one interviews (three interviews being conducted via video call and 22 by telephone) and four focus groups (*n* = 22) via online video call. Combining different qualitative methods within a single study is a common approach used to enhance the robustness of data and ensure findings are comprehensive and well-developed [15]. Collecting data using both one to one interviews and focus groups was important in gaining a more comprehensive understanding of the impact of COVID 19 within the Muslim community, by expanding both the breadth and depth of findings [16]. For example, one-to-one interviews enable detailed exploration of personal experiences of the pandemic, whilst focus groups (through interactions and discussion between participants) provide rich information about the range of perspectives and experiences and facilitate exploration of points of similarity and difference in opinion and beliefs about COVID 19 measures and their impact [16].

An interview guide was developed by the research team [SH, AR, NT, MG] and included semi-structured questions with prompts to facilitate discussion [14]. This guide was linked to that used for the wider household, community and employer study. The topic guide included open questions such as ‘what has been challenging for you during COVID’, ‘how did you find the current restrictions that have been introduced such as social isolation’, ‘was there any religious and/or cultural practice that could have influenced your response to COVID-19’, [14].

### Data analysis

We employed thematic analysis as a framework to interpret the study’s findings [17]. Using this approach the research team [SH, AR, NT, MG] initially blind coded transcripts and then worked together to identify codes and create overarching main themes. Informed by symbolic interactionism [18] and social constructionism [19], researchers sought to elucidate the meanings participants ascribed to covid-19 virus and health protection recommendations (social distancing and social isolation). We also sought to understand how these meanings were constructed, by paying attention to participants’ interactions with the world around them and the people and objects within it and their interpretation of these, in the context of the pandemic.

The initial themes identified were explored further with focus groups data and any new themes were added to the developing framework. The initial themes from both data sets were then revisited as a whole and main overarching themes and sub-themes identified.

### Public involvement

NT is a public adviser with ARC NWC and a member of the study team. NT reviewed study design, supported initial contacts and links to the Muslim population, undertook some data collection, and contributed to the analysis, and writing of this paper.

### Research ethics approval

This study has been reviewed and approved by the University of Liverpool ethical committee [Reference number: 7685].

## Results

### Sample demographics

A total of 47 participants took part in the study. Among these 25 participants (12 Males and 13 females) provided one to one interviews, ages ranged from 19 to 65 years (Mean: 37.5). The number of people living within households ranged from 2 to 9 and the majority of households (18) had at least one key worker (as defined by the participant) living in the household. The majority of participants (17) were in paid employment, five of whom were self-employed. Five participants were in higher education, and three were not in paid employment.

A total of 22 participants attended one of the four scheduled focus groups. Specific participant demographics were not collected due to the setup of focus groups, however, there were 10 male and 12 female participants with ages ranging from 18 to 60 years of age and the sample included representation from people who were employed, unemployed or in higher education. Ethnicities of participants in both interviews and focus groups as defined by themselves were: British Arab, British Yemeni, Arab, British Pakistani, Indian/Asian/Bangladeshi/Pakistani, White European, Black British, Somali, and Black African. The Muslim population is a more diverse community than usually assumed.

#### Qualitative findings

We explored participants’ experiences of COVID-19 and the restrictions to slow transmission, by focusing on the meaning participants attached to the virus and imposed restrictions. Participants’ interpretations of COVID-19 and health protection measures were given meaning and interpreted through their interactions with other people and objects situated within their every-day life. Whilst all households have likely been impacted by imposed restrictions on social interactions, some of those restrictions (and their consequences) in the current sample, impacted social, cultural and religious interactions and practices that are of specific importance to members of the Muslim community.

Five main themes were identified in the data that exemplified the impact of COVID-19 and imposed restrictions on the lives of members of the Muslim community: 1) Psychological distress; 2) The impact of imposed restrictions on fundamental interactions; 3) Challenges during lockdown 4) Religious beliefs as mediators of psychological distress 5) Positive impacts.

#### Psychological distress

This was an overarching theme with references to psychological distress permeating participants’ narratives about COVID-19 and the restrictions imposed to slow its spread. ‘Worry’ (and other similar descriptors e.g. ‘anxious’, ‘scared’, ‘afraid’, ‘panic’) was a widespread feature of participant’s narratives about the virus.

*The first three weeks of the lockdown people were very anxious … I was so worried I felt like if I was going to die from being worried so much about myself and I didn’t know how, what to do [P6]*

Whilst participants were concerned about contracting the virus themselves, they were more concerned about passing the virus on to others, particularly family members and those more vulnerable within society. These concerns were heighted by the sense of responsibility to others instilled within the Muslim community.

*It has been a lot of like worry and panic with it … Because if like I bump into someone whose got it then and I bring it home that’s you know 8 other people that I can infect and my mum’s health isn’t good [P7]*

A number of participants spoke about others’ fears, suggesting that their fear of contracting the virus was so great, that they *‘overdid the caution’*. Others spoke about how they felt the virus had exacerbated pre-existing anxieties for some, and created new anxieties for others.

*I have had people going to the extreme of washing every single item of their shopping when they bring it back to the house. Which is something I've never heard this before this COVID thing. I've heard it with OCD people but not in like your day to day kind of normal person [P21]*

Participants’ concerns about contracting the virus were highlighted due to the growing evidence of increased risk for people from Ethnic Minority communities. As participants were unclear based on current evidence, as to why people from Ethnic Minority communities were at higher risk, their concerns related to the risk associated with underlying health conditions.

*I’m [age 40-60] year old so I have diabetes as well. So I am really concerned I’m very careful because I have diabetes. So that’s why I think I’m very, very scared actually my manager called me, I was actually [company name], those people called me for work but I just said I can’t come because my condition, my age... So I feel not safe. So that's why I did not start work [FG2]*

Participant’s concerns (and their perceptions of other’s concerns) about contracting the virus, were often based on evidence drawn from their own experience, for example, one participant spoke about their awareness of the anxiety of colleagues through the behaviour of workmates towards the participant – distancing themselves from them because they travelled to work on public transport.

*It’s [avoiding public transport] not only for me but also my staff over there my colleagues in my workplace. They have at one time, I was seeing their anxieties and stress because they were avoiding a few things when I was around, so I think it [staying locally and not taking public transport] brought their anxieties down as well as mine [FG2]*

Those working directly with patients and members of the public had particular concerns about contracting the virus. Inadequate provision of personal protective equipment (PPE) during the early phase of the pandemic was a key concern for some. One participant, situated this issue in the context of their contractual obligations, which they interpreted as meaning they were powerless to protect themselves from risk.

*no one of the staff have adequate protection we’re all helpless and you have to see the patients [P8]*

Another participant situated their concerns within the context of their employment status, their current lack of access to financial or social support and responsibility for dependent children. As such, it was the potential consequences for their children, rather than for themselves that was the source of their distress.

*Like self-employed they don't really get much support … ..until now we get no support from government even we applied so we have no choice just to work and when you work you worry might get sick. What will happen because have no like family close like my mum or dad here to look after kids if anything, if we end up in hospital what would happen, things like this you're over thinking and your worried [P11]*

As well as concerns about contracting the virus, participants also spoke about the psychological distress associated with the imposed restrictions to reduce spread of the virus.

#### The impact of imposed restrictions on fundamental interactions

Participants described how restrictions impacted on fundamental human interactions that were central to family, community, social and religious life. These interactions were described by participants as integral to their *sense of belonging* and *connectedness* within the community and consequently restrictions on these interactions contributed to psychological distress.

*We especially as Muslims as Ethnic Minority, we thrive on communities … these communities makes who we are trust me it isn’t easy to completely to take that away, I’m feeling lost … . because those gatherings [In Mosques, community study groups, community centres, Islamic activity groups etc.] bring a lot of Barakh [blessing] in there and its that’s sense of belongingness as well that’s all gone [P1]*

Lockdown restrictions impacted on these types of interactions both through closure of key social spaces and cessation of related activities (e.g. Mosques, community centres) and due to restrictions place on interactions between different households that were part of normative religious practice. Restrictions falling across the month of Ramadan and the Eid festival meant that families and communities were unable to come together to break the fast and experience that sense of connection to one another, which was a source of considerable distress.

*Normally we invite my mother in law, my sister in law to break the fast with us, so it make us feel more of a family occasion and more of connection to each other which is very important in our faith especially with the relatives we weren’t able to have that … it’s very hard [P3]*

All participants spoke about the significant role that religion played in their daily life and the impact of restrictions on the way religious practices were being done, here again restrictions meant changes to fundamental interactions, for example, not being able to take part in community worship which is central to the Muslim faith. Whereas anxiety was associated with the virus, the restriction imposed on these fundamental interactions were associated with a sense of sadness and low mood.

*I know a lot of my friends were you know still living in their university households and self-isolating by themselves so Ramadan was obviously it’s nothing like previous years but it was very, very different for them. Not really community feel and I know a lot of people are feeling quite low about it [FG3]*

Mosques were identified as central in providing not only spiritual, but also social, emotional and in some instances even financial support. Their closure therefore whilst supported, was considered to be particularly challenging and described as having a detrimental impact on the wellbeing of the community, particularly those for whom the mosque was their main if not only source of social contact.

*Mosques where people used to come together and as well as exchange information but also there used to be food in the Mosque … money from the worshippers … all kind of stuff, all of that has just completely gone … how important they are to the community in terms of mental health, how actually important they are in terms of relieving person of stress and things like that … .because when people come there's a lot of benefits, people coming and talking to people and definitely the Mosque was anti-isolation [P12]*

Older members of the community who were self-isolating were described as experiencing particular difficulties, with the sense being, that they were losing connection with the wider community due to closure of the mosques where people felt ‘connection’ with others within their community.

*And one of the things I’m hearing ‘I can’t go out you know I used to enjoy going out and I used to’, especially the elderly, everyone used to go out and they used to go to a mosque you know and ‘I cannot do that now’ and you know I've got my mum now and she said ‘people bring me the food but I don't have that you know personal connection with people’ [P12].*

Similarly, participants described how their families were struggling with being socially distanced from each other, with older family members in particular struggling with this separation;

“*they [parents] feel so lonely and they say ‘whenever you can you can come’ I don't come that often” [P6]*

Distancing restrictions also had implications for normative social conventions including extending hospitality within one’s own home to those in need, which was a further source of distress for some participants, who felt they were failing in their ‘duty’ to others.

*if they’re living on their own you feel as if you have to give them iftar [food to break the fast], bring them over, let them feel that sense of love that sense of, we’re here you’re not alone. We can’t even invite my neighbour she’s quite elderly as well and she’s got issues … but I feel like I’m being bad for not letting her come over … I feel like I’m not doing my duty [P5]*

Participants described their interactions with others as very ‘tactile’, with hand shaking, hand kissing, head kissing and hugging being normative practices that conveyed warmth, respect and closeness within the community, having to refrain from such practices felt ‘cold’ and could be perceived by some as offensive.

*Then you feel embarrassed if someone just reached the hand just to shake it and just say I mean it’s, it’s a bit weird to say no its coronavirus. Some people accept it, some people don't you know … I mean just they feel offended or something, this ‘he doesn’t want to shake my hand. I’m not with coronavirus’ [FG1]*

The requirement to socially isolate if symptoms were experienced was a source of distress for some participants. Distress was associated with the threat of having to isolate for 14 days in the context of work and the potential consequences of this, and also being isolated from other people during a time of need.

*The self-isolation is the most distressing thing … this the one that distressed me doing this at the time … not being able to see for example if one of my family is infected not me being able to visit them and see them and this is a difficult time or for them not being able to see me [P6]*

#### Challenges during lockdown

Participants described a number of different challenges during the initial lockdown when restrictions were being implemented and those on the frontline were working to adopt changes to the way they worked. Implementing health protection measures within their businesses or at their places of work was stressful, particularly so in the early days of the pandemic when there was no specific guidance for smaller independent business.

*It’s the stress of worrying about the staff, of not knowing what to do. When it started we didn’t know anything, we didn’t know how to take the precautionary measures especially because we are a small independent [business] we don't have like an advisory team [P3]*

Participants also spoke about other challenges related to information provision and guidance including, keeping up to date and respond both in work and at home, to the continually changing guidance.

*I started working in my trust we were set up with a new email and get three or four emails every single day … and you have to read them because it’s changing sort of their policies and guidelines every single time … and you come in the next day and someone’s like oh you're not wearing this at this time or you're not doing this correctly and everything’s new now you have to keep up to date with it [FG3]*

The frequency of changes to guidance led some people to question the validity of the guidance and as one participant described, left them feeling that there was no benefit in following the guidance.

*Seem like there is no absolute truth … I’ve heard the coronavirus maybe like in the air for 9 hours … it does change every time at the beginning they said its 15 minutes, so I tell myself if I am shopping, in 15 minutes I leave everything outside, then they start changing this information, so I tried to follow all the information at the beginning but then when I found these instructions it could be like 12 hours, I found that there’s no benefit from following the instruction [P19]*

The impact of lockdown for those who are self-employed and in jobs directly impacted by restrictions was highlighted. The potential for job loss appeared significant and there was particular concern for members of the community on low wages and not eligible for statutory support.

*The community is quite depressed in terms of jobs and the way things are … ..a lot of people are taxi drivers, people who are self-employed and things like that. I think one thing that really hit the community now is actually a lot of them are now going to lose their jobs or their livelihood because of the fact that they have to shut down … so that also has created a lot of mental health [P12]*

Finally, whilst most participants described how they had accepted many of the necessary changes, particular adjustments (e.g. having to change the wearing of a head scarf, having to shave off a beard to ensure correct fitting of PPE and not being able to pray in the work environment) were experienced as distressing, as they were interpreted by some participants as ‘compromising’ their faith.

*I can’t wear my Hijab [head covering] properly I have to wear like a bonnet because then it is easy to transmit [COVID] that way. Therefore, you start feeling, am I in the right job, do I compromise my religion because of my job and then it starts affecting you psychologically because if you’re a practising person yourself the fact that ok how do I do this, where do I find the balance [P1]*

#### Religious beliefs as mediators of psychological distress

Whilst many participants spoke about the psychological toll imposed by COVID-19 and the imposed restrictions, some also described how their religious beliefs and teachings were helping to ameliorate their distress. These different beliefs and teachings appeared to function in a number of different ways to help people cope during the pandemic.

##### Belief in fate and death as Allah’s (god) decree

Participants described how people in general were fearful of dying due to COVID-19, but due to their belief in fate and that death is in Allah’s written decree for them, they themselves did not fear death. Because of this belief participants felt they could only do what they could to try to prevent transmission of the virus and place their trust in Allah that what is meant to be will be, which was said to have a ‘calming’ effect.

*I would say religion has a calming effect on us, to believe in destiny and in the hands of God, we do what we can and the rest is in the hands of God. So that I think has a good psychological effect and in turn has a good effect on our immune system [P24]*

##### Belief that COVID-19 is a test

There was also a belief that COVID-19 was a ‘test’ – there was a lesson to be learnt – everything that happens Allah has a wisdom to it. This idea of purpose was important since it provided a reassurance that there is *‘always a meaning behind calamity’* [FG3] consequently, their faith was a *‘really useful coping mechanism’* [FG3]. Participants also described how through being tested they would be divinely rewarded by Allah - either if they contracted the virus and/or die from it, or if they showed patience during the pandemic. Because of this, one participant described such consequences as a ‘*win win situation’*.

*To me it’s a test and if it’s a test it has an end and Inshallah [if God wills] the outcome and the ending is a better one [P1]*

##### Coping through prayers and supplication

With the belief of COVID-19 as a test from Allah, participants believed that should one be tested, they would be able to seek strength and cure from Allah and they described how prayers and supplication upon Allah helped them cope when experiencing COVID symptoms.

*I used just try and read or listen to the Quran with my Ipad and my kids who are like [under 11 years old]. They started taking turns and just like one after the other they just used to come in my room sit by me and just read it because I used to tell them just read it and Allah will help us. Although we were scared we had this contentment and peace and we were very serene [P8]*

#### Positive impact of COVID-19

Finally, although most participants spoke of the negative impacts of COVID-19 and the restrictions put in place to slow its spread, some also spoke of positive consequences. For example, the requirement to stay at home meant not having to go through the hustle and bustle of the daily routine of getting children to school or the commute into work. This meant there was more space and time for family.

*We are very close now, which we were not, and that we know the nature of our children, our parents, other relatives and relations. So this is a very good lesson for all of us. I know, I don't know loads of things about children which I (laughs) I discovered during lockdown (FG4)*

Participants also reported how the month of Ramadan was a time for community worship, however, due to the restrictions participants instead were staying at home and praying together as a family – bringing them ‘closer’.

*It would have brought probably families closer because I know in our household, especially, we started praying together more often, we started sitting down and reading the Quran together and we started doing, especially because my father’s not going out outside for work as often its there's a lot more interaction within the family, so it does bring us closer as well in some ways. While it is distancing us from other things [P18]*

Some also spoke about how having been relieved of the distractions of working outside the home, they had found more time for spiritual practices.

*This was one of the best Ramadan in terms of spirituality because I was not rushed, I didn’t have to go to work, I work from home and I did my Salah [prayer] the five Salah [FG3]*

For one participant the restrictions had relieved them of some social obligations (regular visits to extended family), which they usually had to fit into their busy working life, the restrictions meant that there was ‘understanding’ that this was not now possible, whilst also making more time for themselves.

*Obviously you feel guilty and you want to go and see your parent and your in laws, you feel like you haven’t seen them for a while. Obviously life’s busy because of coronavirus everyone understands [P4]*

## Discussion

The central theme running through participants’ narratives about the impact of the pandemic was one of psychological distress, with participants describing feelings of anxiety, sadness and low mood either experienced by themselves, their family members or within the wider community. This finding is consistent with recent literature reviews exploring impact of COVID-19 [20, 21] and psychological distress during COVID-19 pandemic that identify anxiety, depression and stress as common experiences [22, 23]. In our study psychological distress was associated both with the virus itself such as concerns about contracting the virus and passing it to other people, and lockdown restrictions. A study conducted in China during the pandemic has reported similar findings [22].

Several participants described excessive hand washing (e.g. every few minutes, every half an hour or ‘hundreds of times’ a day) and the consequences of this for the physical condition of their hands. Several also provided example of how worries about contracting the virus are exacerbating pre-existing tendencies of some people towards obsessive compulsive behaviours. This finding supports recent evidence of impact of the pandemic on obsessive compulsive behaviours [24, 25]. In our study, some participants suggested that worries about contracting the virus were not only exacerbating pre-existing OCD behaviours but also contributing to new incidences of obsessive compulsive behaviours. Our findings in this respect support a recent commentary by Shafran et al. [26] suggesting that even people with no pre-existing concerns can ‘fall into the trap’ of compulsive handwashing because of the relief from anxiety this behaviour can afford, implicating ‘repeated, stereotypical and timed hand washing processes’ advocated during the pandemic as contributing to new incidences of OCD behaviours.

Our findings also highlight the psychological consequences of restrictions put in place to control the spread of the virus. Lockdown restrictions had a particular impact for those who were self-employed and unable to access statutory benefits and there were fears that there were many job losses to come due to the types of employment of many members of the Muslim community, many of whom are self-employed. Impact on employment, was predicted by participants, to contribute to substantial mental health difficulties within the community in the future. It is of note that a survey by Mind, the mental health charity, highlights how existing inequalities experienced by Ethnic Minority communities, including in employment, have had a greater impact on the mental health of Ethnic Minority populations than white populations during the pandemic [27].

There was also recognition of the psychological burden faced by those on the frontline who were more exposed to the virus [14]. Here psychological strain was multifactorial and included, concerns about passing the virus to family members, pressure of implementing regulatory guidance, and, seeking to keep both themselves and their clients/customers safe, which was noted as particularly stressful for those working in independent business who received little or no guidance early on in the pandemic.

Restrictions on human interactions was also identified as a source of considerable distress. Whilst the impact of restrictions on human interactions across the UK population as a whole has been well documented [28] for members of the Muslim community there were specific consequences of restrictions on fundamental cultural and religious practices. The initial lockdown period from March to May 2020, saw the closure of Mosques and restrictions on people gathering together with specific consequences for members of the Muslim community who were unable to come together as a community to break the fast in congregation at the end of the holy month of Ramadan. Such practices are integral to individual sense of belonging and connectedness within the Muslim community. Social connectedness has long been recognised as important for psychological wellbeing and a recent survey undertaken during COVID-19 ‘stay at home’ measures in Austria, highlighted the relationship between social connectedness and psychological wellbeing [29]. Our findings provide some insight with regard to this relationship, indicating that it is the specific meanings that people ascribe to human interactions that influence experiences of psychological distress.

The closure of Mosques (and consequently cessation of community prayers), was identified as a particular challenge for the Muslim community, due to the sense of spiritual togetherness such interactions bring. Lockdown restrictions also effected other faiths such as the Jewish Hindu and Buddhist communities whilst for Christians, restrictions were briefly eased for Christmas 2020 so that families could meet together in bubbles to celebrate together [30]. Should the current national lockdown restrictions continue, community worship and celebrations including Ramadan and Eid 2021 may again be disrupted with the potential to further impact mental wellbeing within the Muslim community.

Mosques were not only places of spiritual togetherness, they also provided community members with practical, social and emotional support. Even though the closure of the Mosques were consider appropriate by participants, this had implications for individual wellbeing. This was deemed to be particularly so for older members of the community and there were concerns that closure of mosques might have left some within the community isolated and at risk of mental distress. It has been reported previously how social isolation can lead to a ‘deep disconnection’ [31] for those who live alone and are unable to get adequate support, increasing the likelihood of depressive symptoms, which may extend well beyond the end of the current pandemic [31, 32]. Some of the common aspects of psychological distress recognised as consequences of prolonged social isolation (such as anxiety, panic and obsessive-compulsive symptoms) [32] are present in the narratives of participants in the current study. Even short periods of social isolation (less than 10 days) have been reported to have long term (up to 3 years) psychological effects [33]. A recent UK-based study found that self-isolation prior to lockdown, increased feelings of isolation during and since lockdown [34]. It is perhaps not surprising then that participants in the current study report psychological distress of families, particularly those older members, separated not only from family members living outside of their households but also from other members of their community due to the disruption of usual mechanisms of social and emotional support, provided by Mosques or other community-based organisations.

Furthermore, despite the technological benefits of social media platforms that enabled people to keep in contact, such platforms cannot substitute for the physical closeness that is integral to interactions within a highly ‘tactile’ culture. Participants in our study described how challenging it was not be able to have physical closeness with others, particularly at a time when many may be in need of comfort – so often conveyed in physical ways such as hugs or welcoming someone into their home to share their hospitality. It has been said previously that the very ‘nature’ of human beings makes it unlikely that we will manage long-term segregation well [31]. In our study the lack of physical closeness due to COVID-19 restrictions was described as leaving some people feeling a lack of connection with their community. There were concerns that current restrictions could have a long-term effect on human interaction as people come to perceive their proximity to others as potentially threatening [31]. These long-term effects were a concern for participants in the current study, with growing evidence that new ways of interacting (that are less tactile) were becoming accepted and had the potential to gradually replace the traditional old worldview of interpersonal relationships [31].

Whilst participants spoke of the considerable stress and distress experienced by members of the Muslim community as a consequence of the pandemic, they also spoke about how their faith was helping them to cope. Central to this appeared to be the acceptance of the situation as it presented, the belief that whatever happened was ‘meant to be’ and therefore, ‘made sense’, and the belief that death was ordained by Allah and therefore was not something to be feared. Our study’s findings reflected what has been described as ‘positive religious coping’, which includes being adaptive and sense-making based on reflection on the meaningfulness of life and depending on a secure relationship with a merciful God [35].

These findings also reflect those of a recent survey [36] that explored positive religious coping, depression and anxiety symptoms and history of psychological disorder amongst Muslims and Christians in the United Arab Emirates during the early stages of the national response to the COVID-19 pandemic. Authors reported that Muslims described significantly higher levels of ‘positive’ religious coping, with positive religious coping being inversely related to depressive symptoms and history of psychological disorder. They concluded that ‘positive religious coping during infectious disease outbreaks may help some individuals reduce their risk of depression [36], which may be of potential benefit for the Muslim community in the UK.

Evidence from our study and those of others suggest that further research is needed to develop a more detailed understanding of the role of culture and faith in coping and recovery of populations during times of pandemic and the importance of consideration of these when developing COVID-19 guidance [37, 38]. Whilst closure of Mosques were welcomed to control the spread of the virus, such closures had an impact on usual mechanisms for conveying important information and providing spiritual, emotional and practical support within the Muslim community. It is important that during times of crisis such as a pandemic, alternative mechanisms including online congregation and alternative sources of support are made available. Collaboration with faith leaders is essential to ensure key messages and support continue to be provided during such times.

### Strengths and limitations

This is one of the first studies to conduct an in-depth exploration with members of the Muslim community about their perceptions and experiences of COVID-19 and the pandemic. A key strength is that both the researcher that conducted the interviews and the Public Adviser who co-facilitated focus group discussions were from a Muslim background, this was important for facilitating discussion and for participants to express their experiences in ways and through language universally familiar within the Muslim community.

The main limitation, as with many research studies, was that we were only able to explore experiences of those people who wished to take part in the study and consequently our findings may not represent all sections of the Muslim community.

Through our recruitment strategy we sought to ensure representation from across the age range within the Muslim community including those over the age of 65 (given that those over 65 have been found to be at increased risk from COVID-19). Potential participants were contacted through key contacts within the community and through the study’s public adviser. In spite of our attempts to include older members of the community in our sample, we were unable to recruit any participants over the age of 65 years, however some participants reported views held by older members of their family and more broadly in the wider community. This offered second-hand insight into the experiences of older members of the Muslim community. The lack of over 65 year olds may be due to languages barriers within this age group, further research should therefore seek to capture the experiences of non-English speakers.

## Conclusion

Psychological distress was associated with COVID-19 virus and impact of COVID-19 restrictions on livelihoods and fundamental human interactions. Faith provided an important foundation for Muslim individuals’ resilience. Better provision of culturally and religiously appropriate information, improving local channels of communication (e.g. through faith leaders) and provision of practical support are important during times of pandemic when usual mechanisms of support may be disrupted.

## Data Availability

The datasets generated and/or analysed during the current study are not publicly available as they may contain information that could compromise the confidentiality and anonymity of the participants but are available (limited) from the corresponding author on reasonable request.
